# E3 Ligase Ligands for PROTACs: How They Were Found and How to Discover New Ones

**DOI:** 10.1177/2472555220965528

**Published:** 2020-11-03

**Authors:** Tasuku Ishida, Alessio Ciulli

**Affiliations:** 1 Division of Biological Chemistry and Drug Discovery, School of Life Sciences, University of Dundee, Dundee, UK

**Keywords:** PROTACs, E3 ubiquitin ligase, targeted protein degradation, binding ligands, molecular glues

## Abstract

Bifunctional degrader molecules, also called proteolysis-targeting chimeras (PROTACs), are a new modality of chemical tools and potential therapeutics to understand and treat human disease. A required PROTAC component is a ligand binding to an E3 ubiquitin ligase, which is then joined to another ligand binding to a protein to be degraded via the ubiquitin–proteasome system. The advent of nonpeptidic small-molecule E3 ligase ligands, notably for von Hippel–Lindau (VHL) and cereblon (CRBN), revolutionized the field and ushered in the design of drug-like PROTACs with potent and selective degradation activity. A first wave of PROTAC drugs are now undergoing clinical development in cancer, and the field is seeking to extend the repertoire of chemistries that allow hijacking new E3 ligases to improve the scope of targeted protein degradation.

Here, we briefly review how traditional E3 ligase ligands were discovered, and then outline approaches and ligands that have been recently used to discover new E3 ligases for PROTACs. We will then take an outlook at current and future strategies undertaken that invoke either target-based screening or phenotypic-based approaches, including the use of DNA-encoded libraries (DELs), display technologies and cyclic peptides, smaller molecular glue degraders, and covalent warhead ligands. These approaches are ripe for expanding the chemical space of PROTACs and usher in the advent of other emerging bifunctional modalities of proximity-based pharmacology.

## Introduction

Selective modulation of targeted proteins with small molecules is a major strategy to treat disease. In the early 2000s, almost all pharmaceutical companies invested heavily in efforts to develop small-molecule protein modulators, mainly inhibitors, with desirable properties in terms of efficacy and safety. Although a lot of new molecular entities (NMEs) were launched as a result, numerous proteins remain poorly tractable and so challenging to tackle by small molecules. New modalities, such as antibody and oligonucleotides, opened a door to address some of those more challenging targets, but face other limitations such as poor cell permeability and/or chemical instability. Alternative small-molecule modalities are therefore required to expand the range of proteins being targeted for drug discovery.

**Figure 1.**
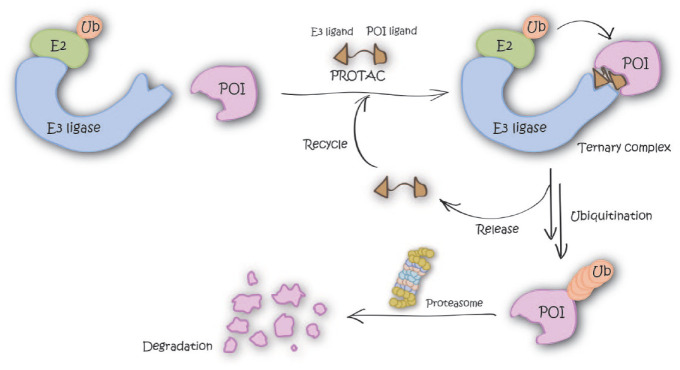
Graphical representation of the degradation mechanism of proteolysis-targeting chimeras (PROTACs).

Inducing degradation of target proteins by bifunctional small molecules, so-called proteolysis-targeting chimeras (PROTACs), is one of the most exciting such new modalities. PROTACs consist of a ligand for recruiting a target protein of interest (POI) and a ligand for an E3 ubiquitin ligase, joined with an appropriate linker. PROTACs induce proximity between an E3 ligase and POI in the form of a ternary complex that results in POI ubiquitination and subsequent degradation by the proteasome (**Fig. 1**). Compared with classical inhibition by small molecules, PROTACs offer several potential advantages: (1) PROTACs are expected to exert similar phenotypes to those observed via knockdowns using genetic tools, such as small interfering RNA (siRNA), short hairpin RNA (shRNA), or clustered regularly interspaced short palindromic repeats (CRISPR), because the downstream result is the same in all those cases (i.e., depletion of intracellular protein levels). Elimination of POI could give additional effect by disrupting formation of biologically functional complexes. (2) PROTACs can work catalytically (i.e., can be recycled so that one PROTAC molecule can turn over multiple molecules of POI) and so can act “sub-stoichiometrically” (i.e., at fractional occupancy of the POI). As a result of this, PROTACs often show higher POI degradation than expected based on their binding affinity to the POI alone. (3) Target protein degradation by PROTACs can suppress resistant mutation and/or upregulation of POI.

In 2001, Sakamoto and coworkers reported a first PROTAC to degrade methionine aminopeptidase (MetAP),^1^ and in 2004, Schneekloth et al. described peptidic von Hippel–Lindau tumor suppressor (pVHL) protein-based PROTACs, which showed cellular permeability and degradation activity against FK506 binding protein 12 (FKBP12) and androgen receptor (AR).^2^ These first-generation PROTACs consisted of a peptidic ligand for an E3 ligase. Peptide moieties caused limited cell permeability, synthetic tractability, and biological instability, which motivated efforts to develop more drug-like, nonpeptidic E3 ligase ligands. During the past few years, these efforts have resulted in improved small-molecule-based PROTACs recruiting cereblon (CRBN),^3^ von Hippel–Lindau (VHL),^4^ and inhibitors of apoptosis proteins (IAPs).^5^ By leveraging these small-molecule E3 ligase ligands, the field has since extensively demonstrated that PROTACs can induce degradation of a variety of proteins, even at sub-nanomolar concentrations, and has validated their applications not only as biological tools but also as a new chemical modality for treatment of diseases in the clinic. Moreover, modern medicinal chemistry efforts have enabled the development of PROTACs with acceptable drug-like properties. In 2020, Arvinas presented interim results of a Phase 1/2 clinical trial of their front-line PROTAC, ARV-110, in men with metastatic castration-resistant prostate cancer (mCRPC) and showed two patients achieving responses in prostate-specific antigen (PSA) levels.^6,7^ This landmark result showed pharmacological efficacy of PROTACs in the clinic. The number of E3 ligases currently being explored by the most advanced PROTAC molecules remains small (typically, VHL, CRBN, and IAPs), however, limiting scope. Expansion of the E3 ligase toolbox will therefore be important not only to facilitate degrading a broader range of proteins but also to potentially induce less systemic and more selective (e.g., tissue- or organ-specific) degradation of target proteins by exploiting differential biology and expression levels of E3 ligases.

Here, we first briefly recount how ligands of the typical E3 ligases for PROTACs were discovered, and then review approaches that have been recently used to discover new ligands and new E3 ligases for PROTACs. We then outline the diverse technologies that, in our opinion, provide the most compelling and suited strategies to find new E3 ligase ligands for PROTACs, including applications of structure-based ligand design, DNA-encoded libraries (DELs), display technologies for macrocyclic peptides, molecular glues, and covalent warhead ligands.

### Discovery of the Traditional E3 Ligase Ligands for PROTACs

#### VHL Ligands and Applications to PROTACs

In 1992, the hypoxia-inducible factor-1α (HIF1α) was found in Hep3B cells as a factor inducing erythropoietin under hypoxic conditions.^8^ After this finding, it was discovered that HIF1α also regulates a variety of biological responses (e.g., metabolic reprogramming,^9^ suppression of reactive oxygen species [ROS] production,^10^ angiogenesis,^11^ epithelial-mesenchymal transition,^12^ and cancer metastasis^13^). Under normoxic conditions, intracellular concentrations of HIF1α are kept at low levels by hydroxylation of specific proline residues and subsequent ubiquitination and proteasomal degradation. The protein responsible for this process was later found to be the pVHL protein, a tumor suppressor protein mutated in VHL disease.^14,15^ pVHL is a subunit of the E3 ubiquitin ligase CUL2–RBX1–ElonginB–ElonginC–VHL (CRL2^VHL^) complex.^16–18^ The cocrystal structure of the pVHL complex and C-terminal oxygen-dependent degradation (CODD) motif of HIF1α revealed the structural recognition of a key hydroxyproline residue in hydroxylated HIF1α by pVHL.^19,20^

Early PROTACs consisted of epitope peptide CODD motif (ALAPYIP) conjugated to a poly-D-arginine tag at the C-terminus, and to a target binding ligand at the N-terminus. These peptidic PROTACs induced degradation of intracellular FKBP12 and AR without need of microinjection, albeit at fairly high concentrations.^2^ The moiety of the peptidic pVHL ligand, however, limited further application as mentioned before; therefore, development of nonpeptidic pVHL ligands was required to overcome this issue.

**Figure 2.**
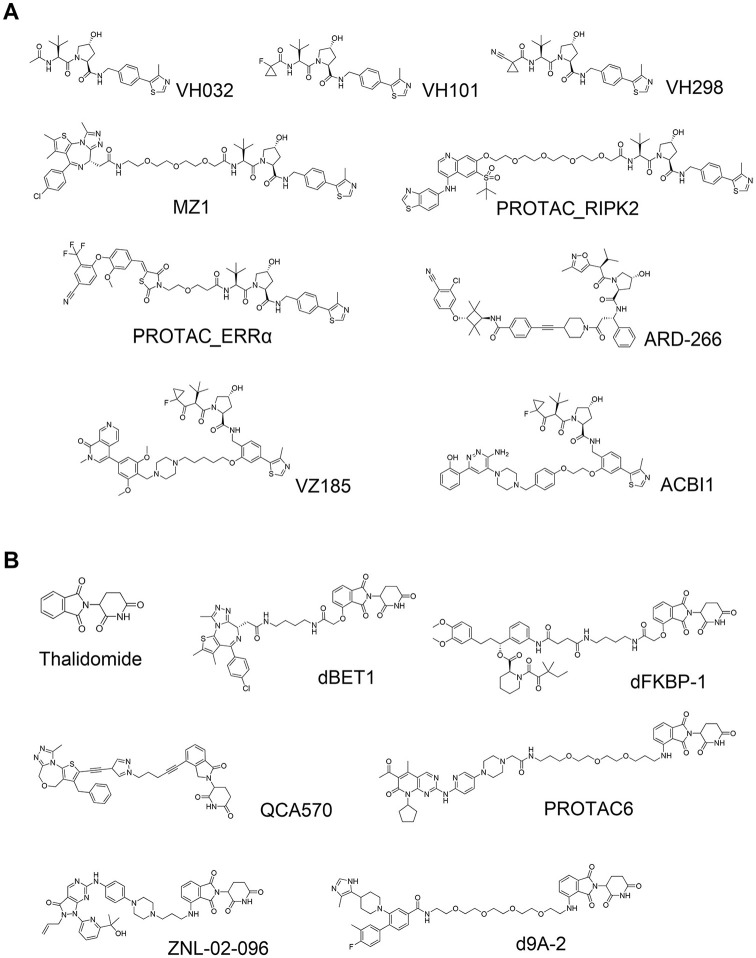
Von Hippel–Lindau (VHL)-based and cereblon (CRBN)-based small-molecule proteolysis-targeting chimeras (PROTACs). (**A**) Exemplified structures of VHL ligands and the PROTACs composed of them: VH032,^24,26^ VH101,^25^ VH298,^25,26^ MZ1,^4^ PROTAC_ RIPK2,^27^ PROTAC_ ERRα,^27^ ARD-266,^137^ VZ185,^77^ and ACBI1.^138^ (**B**) Exemplified structures of thalidomide and PROTACs composed of CRBN ligands: thalidomide,^28^ dBET1,^3^ dFKBP12,^3^ QCA570,^139^ PROTAC6,^140^ ZNL-02-096,^141^ and d9A-2.^142^

In 2012, novel peptidomimetic pVHL ligands were developed by the laboratories of Crews and Ciulli, by leveraging the hydroxyproline core fragment that forms critical binding interactions with Ser111 and His115 in the hydroxyproline binding pocket on pVHL. Computational approaches to design plausible ligands and subsequent cocrystal structures guided rational medicinal chemistry optimization that yielded a first small-molecule pVHL–HIF1α protein–protein interaction inhibitor with dissociation constants of single-digit micromolars.^21–23^ Galdeano et al. later developed second-generation ligands via structure-based drug design using cocrystal structures and isothermal titration calorimetry (ITC) to optimize, in a stepwise rational fashion, interactions at the left-hand side of the VHL ligand. As a result of their optimization study, strong VHL binders with nanomolar binding affinity to VHL were finally obtained, disclosing VHL ligand VH032 (**Fig. 2A**).^24^ Subsequent medicinal chemistry work by the Ciulli group optimizing the “capping group” at the left-hand side of VH032 found that this position is tolerant for and can accommodate a variety of substituents. They identified fluoro- and cyano-cyclopropyl capping groups, which led to novel compounds (VH101 and VH298, respectively; **Fig. 2A**) with sub-100 nM affinity (more potent affinity than a 10-mer HIF1α peptide).^25^ Biophysical and cellular studies qualified VHL inhibitor VH298 as a potent, selective, and cell-active chemical probe of the hypoxic signaling pathway.^26^

The left-hand side of the VHL ligands provided an early suitable exit vector to develop PROTACs. Zengerle et al. leveraged the crystal structure of VH032 bound to VHL to design and develop one of the first VHL-based PROTACs, MZ1 (**Fig. 2A**), which degraded Bromodomain- and Extraterminal domain (BET) proteins BRD2, BRD3, and BRD4 in cancer cells, with unexpectedly selective degradation for BRD4 at single- to double-digit nanomolar levels.^4^ At the same time, Bondeson et al. independently reported PROTACs bearing the same VHL ligand that degraded estrogen-related receptor-α (ERRα) and Receptor-interacting serine/threonine-protein kinase-2 (RIPK2) efficiently both in cells and in vivo (PROTAC_RIPK2 and PROTAC_ERRα; **Fig. 2A**).^27^ These two studies disclosed the first high-quality all-small-molecule VHL-based PROTACs, changing the game in the field and opening future avenues for VHL-based and other E3-recruiting PROTACs.

#### Ligands for CRBN and Application to PROTACs

In 1957, thalidomide (**Fig. 2B**) was launched by Chemie Grünenthal as a drug for insomnia. Shortly following administration and use as a sedative, however, it was found that thalidomide had strong teratogenicity, causing approximately 8000 to 12,000 children to be born with deformities.^28^ After those tragic adverse events, thalidomide was finally withdrawn from the market. Fifty years later, thalidomide was found to have therapeutic effect in patients with multiple myeloma,^29^ but the mechanism of action remained unclear. In 2010, Ito et al. found that thalidomide binds to CRBN, a subunit of the E3 ubiquitin ligase CUL4–RBX1–DDB1–CRBN (CRL4^CRBN^), and CRBN engagement induced at least part of the teratogenic effect in in vivo models.^30^ Two independent groups then separately reported that lenalidomide, a derivative of thalidomide and a marketed drug used for the treatment of hematological malignancies, induced degradation of transcription factor Ikaros family zinc finger protein-1 (IKZF1) and Ikaros family zinc finger protein-3 (IKZF3).^31,32^ In 2014, two X-ray cocrystal structures of DDB1–CRBN–thalidomide complexes were disclosed by the laboratories of Thomä and Celgene. The structures revealed that a portion of the phthalimide ring on thalidomide is exposed to solvent and provided room for extending a substituent or linker.^33,34^ In a manner similar to that pursued with VHL-based PROTACs, Winter et al. and Lu et al. leveraged these findings and crystal structures to develop PROTACs dBET1, ARV825, and dFKBP (**Figure 2B**), which potently and rapidly degraded BET proteins or FKBP12 and demonstrated tumor growth inhibitory activity in cells and in mouse models.^3,35^

#### CRBN and VHL: Key Players in the PROTAC Field

**Figure 3.**
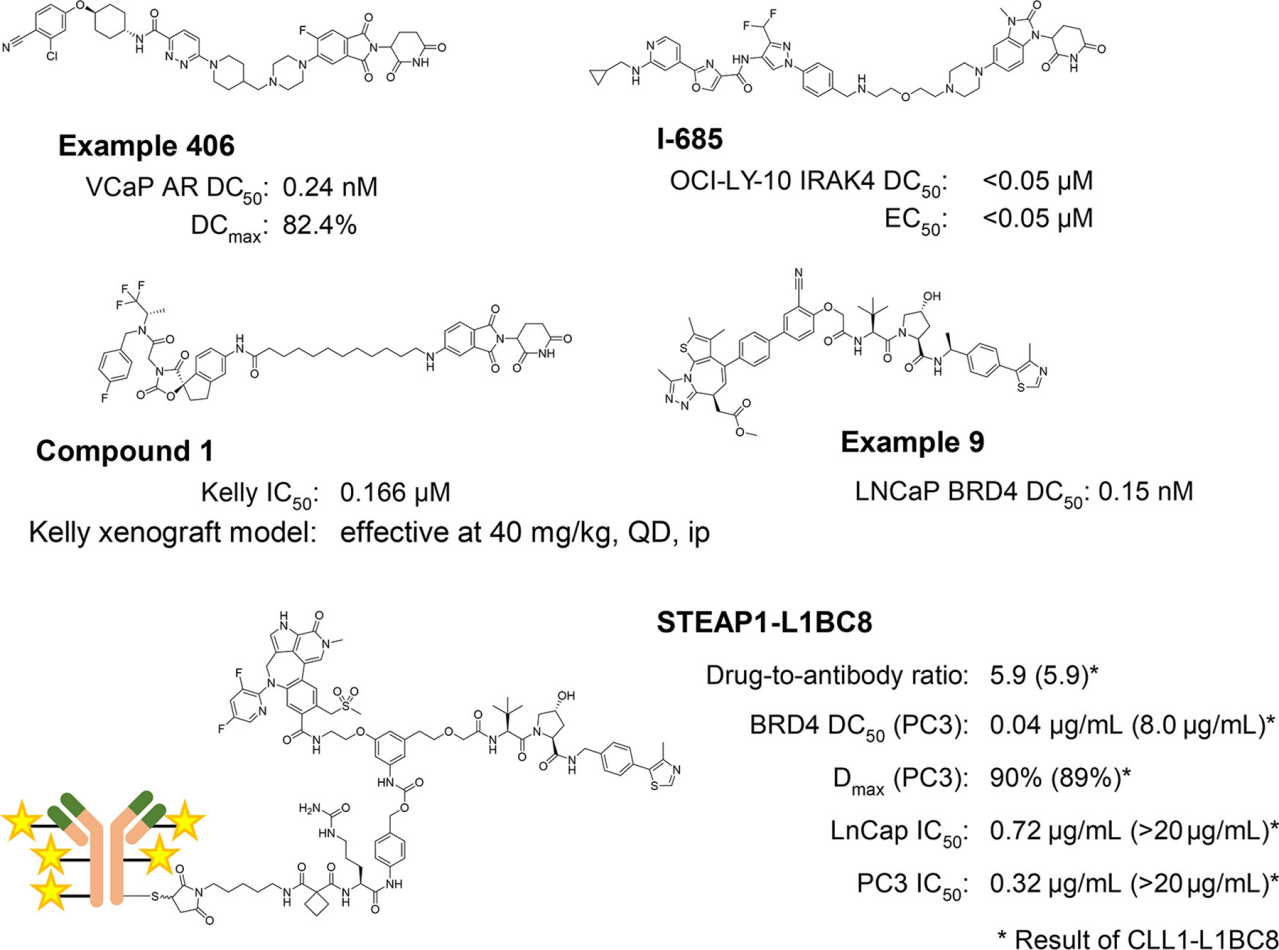
Representative structures and biological data of recently disclosed patents: 406,^143^ I-685,^144^ Compound 1,^145^ Example 9,^146^ and STEAP1-L1BC8.^147^

Currently, the VHL and CRBN ligands are the most popular and most frequently used E3 ligands to design PROTAC degraders. These ligands are the frontrunners in this field because of several favorable features: (1) strong, specific, and biophysically validated binding affinities to their targeted E3 ligase; (2) acceptable physicochemical profile, for example molecular weight, lipophilicity, solubility, lack of obvious reactive groups or metabolic hotspots, and lack of pan-assay interference compounds (PAINS)^36^ alerts; and (3) well-characterized structural information of their binding modes. Because of these properties, numerous PROTACs that are based on CRBN or VHL have been reported that have shown excellent in vitro and in vivo degradation activities for the targeted POI (**Fig. 2**). As a result, PROTACs composed of CRBN or VHL ligand are currently considered one of the most powerful alternative modalities of chemical intervention into biology and promising therapeutics. Therefore, not only Big Pharma but also institutes and biotech firms have filed many patents and are engaged in developing PROTACs all the way into the clinic (**Fig. 3**).

#### PROTACs Based on IAP Ligands

**Figure 4.**
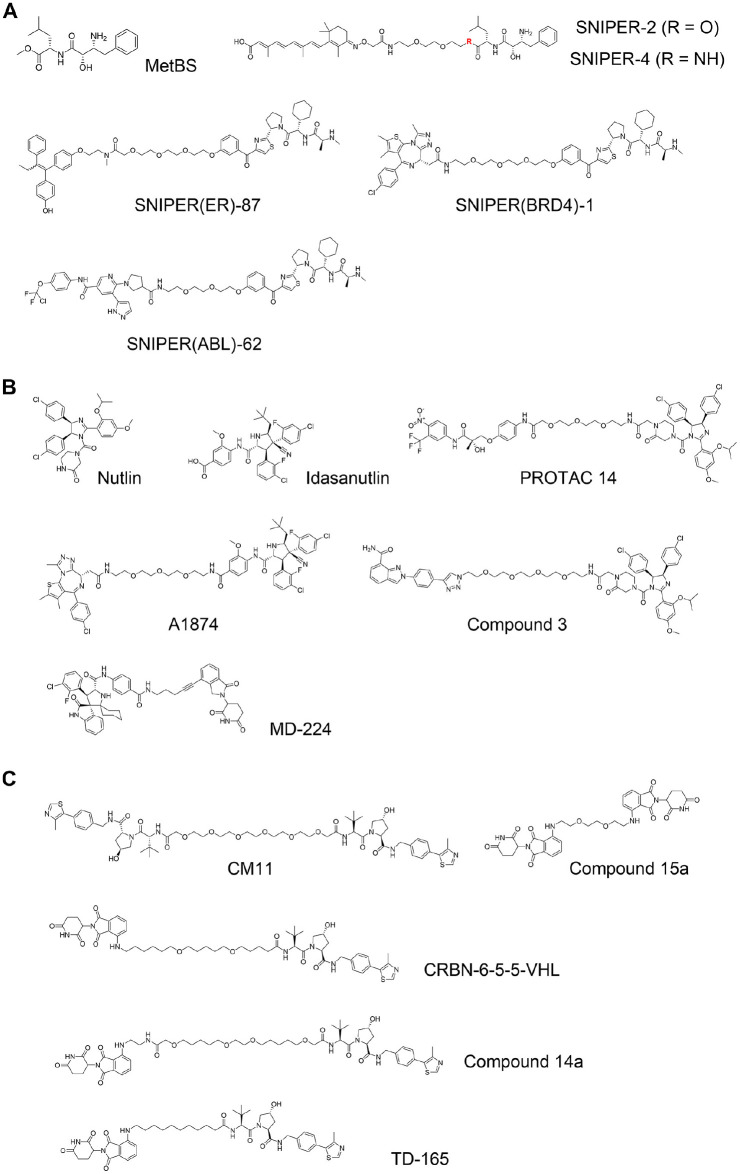
Inhibitor of apoptosis protein (IAP)-based and mouse double minute 2 homolog (MDM2)-based proteolysis-targeting chimeras (PROTACs), and von Hippel–Lindau (VHL)-based and cereblon (CRBN)-based homo-PROTACs. (**A**) Exemplified structures of methyl bestatin (MetBS) and Specific and Nongenetic Inhibitors of Apoptosis Protein (IAP)-Dependent Protein Eraser (SNIPERs): MetBS,^37^ SNIPER-2 (R = O)^5^ and SNIPER-4 (R = NH),^38^ SNIPER(ER)-87,^39^ SNIPER(BRD4)-1,^40^ and SNIPER(ABL)-62.^41^ (**B**) Structures of MDM2 inhibitors and PROTACs incorporating with them: nutlin,^44^ idasanutlin,^46^ PROTAC 14,^45^ A1874,^148^ Compound 3,^149^ and MD-224.^47^ (**C**) Structures of homo-PROTACs and related PROTACs composed with CRBN and VHL ligands: CM11,^48^ Compound 15a,^49^ CRBN-6-5-5-VHL,^50^ Compound 14a,^51^ and TD-165.^52^

The family of IAPs is characterized by three baculovirus IAP repeat (BIR) domains at their N-termini, and has important functions in inhibiting cell apoptosis. Five out of eight IAPs, including cellular inhibitor of apoptosis-1 (cIAP1) and X-linked inhibitor of apoptosis protein (XIAP), also contain Really Interesting New Gene (RING) domains that bind to E2 conjugating enzymes, and so act as E3 ligases. Sekine et al. reported that methyl bestatin (MetBS; **Fig. 4A**) binds to the BIR3 domain of cIAP1 and induces self-ubiquitination following proteasomal degradation of cIAP1.^37^ Structure–activity relationship studies of MetBS suggested that the bestatin portion binds to cIAP1, while the methyl ester is solvent exposed. Based on this observation, bifunctional small molecules were developed that comprise bestatin and all-trans retinoic acid (ATRA) linked by a polyethylene glycol (PEG) linker. These compounds were found to induce degradation of cellular retinoic acid binding protein-2 (CRABP2) (SNIPER2 and SNIPER4; **Fig. 4A**).^5,38^ The authors named this technology Specific and Nongenetic Inhibitor of Apoptosis Protein (IAP)-Dependent Protein Eraser (SNIPER) and later expanded it to other target proteins, such as estrogen receptor (ER),^39^ BRD4,^40^ and BCR-ABL^41^ [SNIPER(ER)-87, SNIPER(BRD4)-1, and SNIPER(ABL)-62; **Fig. 4A**]. One characteristic feature of SNIPERs is that they can retain self-ubiquitination activity toward cIAP1. This effect may show synergistic activity to types of cancer cells in which cIAP1-induced degradation is beneficial. The self-degradation effect, however, reduces intracellular levels of cIAP1 and consequently causes suppression of degradation activity toward the target protein.^42^

#### PROTACs Based on MDM2 Ligands

Tumor protein p53 is a transcription factor that regulates DNA repair, cell cycle, and apoptosis. p53 is mutated in more than 50% of cancers.^43^ Mouse double minute 2 homolog (MDM2) protein is an E3 ubiquitin ligase that induces ubiquitination of p53 and subsequent proteasomal degradation of p53. Nutlin (**Fig. 4B**) is a well-known compound that binds to MDM2 and disrupts MDM2–p53 interaction, and it is among the first small molecules that were discovered to bind to E3 ligase proteins.^44^ In 2008, Schneekloth and coworkers developed a nonpeptidic PROTAC that consisted of an AR ligand and the nutlin motif (PROTAC 14; **Fig. 4B**).^45^ Some more PROTACs based on nutlin or idasanutlin^46^ were later developed (**Fig. 4B**), but the number of MDM2 PROTACs has remained limited, due to their challenging physicochemical profile and limited degradation activity. PROTACs composed of a ligand for CRBN linked to a ligand for MDM2 were found to potently degrade MDM2 protein selectively (MD-224; **Fig. 4B**).^47^ That implies that the degradation efficiency and/or activity of MDM2 is supposed to be limited compared to the Cullin RING ligase complexes formed by CRBN or VHL.

#### Homo-PROTACs

The mode of action of PROTACs toward POIs was expanded by the development of unusual homo-bifunctional molecules that comprised two ligands for the same E3 ligase. Maniaci et al. developed a novel type of PROTACs that were dimers of two VHL ligands joined by PEG linkers, so-called homo-PROTACs.^48^ The best compound, CM11 (**Fig. 4C**), induced dimerization of CRL2^VHL^ with high cooperativity (~20) in vitro and induced intracellular VHL degradation at >100-fold lower concentration of the binary *K*_d_ value. CM11 also induced degradation of CUL2, suggesting a mechanism of bystander ubiquitination and degradation of a subunit different from that being recruited by the homo-PROTAC within the context of the whole CRL complex. Later, Steinebach et al. extended this approach to CRBN and developed homo-PROTACs that degrade CRL4^CRBN^ ligase (Compound 15a; **Fig. 4C**).^49^ Two groups, Steinebach et al. and Girardini et al., then independently reported hetero-bifunctional compounds that are composed with a ligand for CRBN at one end, and a ligand for VHL at the other end (CRBN-6-5-5-VHL and Compound 14a; **Fig. 4C**). Both groups demonstrated that their PROTACs preferably induced the degradation of CRL4^CRBN^ rather than CRL2^VHL^.^50,51^ The same finding was subsequently reported by a third group (TD-165; **Fig. 4C**).^52^ All three groups developed PROTAC degraders of slightly different chemical structures, yet in all cases preferential degradation of CRBN was observed, suggesting that the homo-PROTAC approach could be used to hijack E3 ligases against each other for inducing selective E3 ligase degradation.

### Expansion of the E3 Ligase Toolbox for PROTACs

Currently, PROTAC design and development center on the use of primarily VHL and CRBN ligands, and to some extent IAP ligands. There are, however, more than 600 E3 ligases known to function in human cells, suggesting a large untapped pool of E3 ligases that are potentially hijackable for targeted protein degradation. To expand the scope of the PROTAC modality, efforts to expand the E3 ligase toolbox with novel chemistries have received significant efforts. Next, we briefly highlight the main progress from the past 4–5 years at finding ligands for new E3 ligases.

#### KEAP1

**Figure 5.**
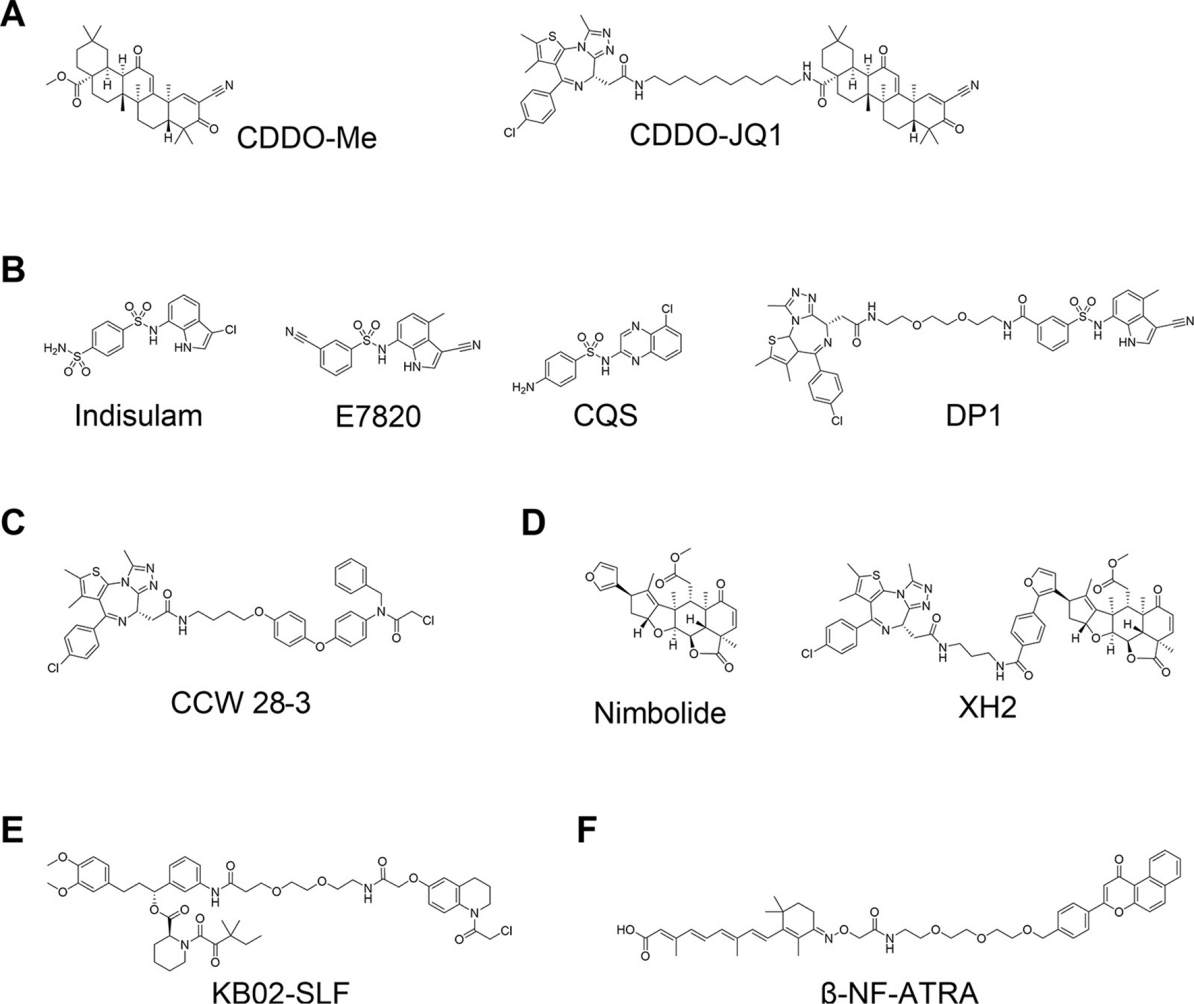
Proteolysis-targeting chimeras (PROTACs) using nonconventional E3 ligase ligands. (**A**) Structures of CDDO-Me^56,57^ and CDDO-JQ1.^66^ (**B**) Structures of sulfonamide derivatives, indisulam,^67^ E7820,^68^ CQS,^69^ and DP1.^78^ (**C**) Structure of CCW 28-3.^79^ (**D**) Structures of nimbolide and XH2.^80^ (**E**) Structure of KB02-SLF.^81^ (**F**) Structure of β-NF-ATRA.^82^

Kelch-like ECH-associated protein-1 (KEAP1) is known to interact with nuclear factor erythroid 2-related factor-2 (Nrf2). The main function of KEAP1 is to regulate cellular responses for chemical and oxidative stress by its role as a substrate recognition subunit of a cullin-3 (CUL3) E3 ligase to bind to induce ubiquitination and degradation of substrate Nrf2.^53^ Academic and industrial groups have throughout the years developed a variety of small molecules that disrupt the KEAP1–Nrf2 interaction based on natural bioactive compounds,^54–57^ high-throughput screening (HTS),^58–60^ virtual screening,^61,62^ or fragment-based drug discovery (FBDD).^63,64^ In 2018, the first PROTACs recruiting the KEAP1–CUL3 E3 ligase were reported that were shown to induce degradation of intracellular Tau at micromolar concentration.^65^ The peptidic nature of the PROTACs, however, which used an Nrf2-based epitope peptide as KEAP1 ligand, limits their potential for expanding their use as in vivo tools for clinical application. Recently, Tong et al. developed the first nonpeptidic PROTACs that consist of JQ1 and bardoxolone methyl (CDDO-Me) as ligands of BET bromodomains and KEAP1, respectively (**Fig. 5A**).^66^ They found that the degradation activity of BRD4 was diminished by removing the Michael acceptor motif of CDDO-Me, indicating that formation of covalent bonds between cysteines on KEAP1 and the Michael acceptor moiety on CDDO-Me is essential to recruit BRD4 to the KEAP1 E3 ligase.

#### DCAF15

Sulfonamide derivatives indisulam (E7070),^67^ E7820,^68^ and chloroquinoxaline sulfonamide (CQS)^69^ were found to have antitumor activity and were tested in the clinic for the treatment of cancers (**Fig. 5B**). Yokoi et al. reported that a cancer cell line resistant to indisulam was also resistant to E7820 and CQS, but not to doxorubicin and paclitaxel;^70^ however, the mechanism of action of those sulfonamide derivatives remained unclear for a long time. In 2017, two groups, Uehara et al.^71^ and Ting et al.,^72^ independently reported that these sulfonamides induce ubiquitination and subsequently proteasomal degradation of coactivator of activating protein-1 and estrogen receptors (CAPERα) via formation of a CAPERα–sulfonamide–DCAF15–DDB1–CUL4 complex. In 2019, three independent groups released cocrystal structures of CAPERα–sulfonamide–DCAF15–DDB1–DDA1 multimeric complexes and confirmed that sulfonamides induce formation of the complexes as a molecular glue.^73–75^ Their structural information could help to further optimize sulfonamides using structure-based drug design and leverage them as DCAF15 ligands for PROTAC design. Until recently, however, it has remained challenging to develop indisulam-based DCAF15-recruiting PROTACs,^76,77^ most likely because of the weak binding affinities of these compounds to DCAF15 alone, or perhaps due to unsuitable orientation between the binding pocket of sulfonamides and the RING domain of CUL4.^74^ In 2020, Li et al. reported a first aryl sulfonamide–based PROTAC, DP1 (**Fig. 5B**), that induces degradation of BRD4 and shows tumor growth inhibitory activity in mouse models in vivo. The biological activity of DP1 is limited, but the results demonstrate that DCAF15 can be applied for development of PROTACs.^78^ Aryl sulfonamide derivatives have a good physicochemical profile (i.e., lower logD values and lower molecular weight compared to most E3 ligase ligands available); therefore, DCAF15 is likely to gain increased attention, and more DCAF15-mediated PROTACs are expected to be developed in the future.

#### Other E3 Ligases for PROTACs: RNF4, RNF114, DCAF16, and AhR

Unconventional approaches have shown promising applications to find new chemistry to expand the repertoire of E3 ligases for developing PROTACs. Ward et al. found novel covalent RNF4 binders by activity-based protein profiling (ABPP)-based screening, and then their optimized ligand was applied to develop a novel type of PROTAC (CCW 28-3; **Fig. 5C**) to target BRD4 for degradation.^79^ Around the same time, Spradlin and coworkers reported that nimbolide (**Figure 5D**), a natural compound that exhibits biological activities in cancer cells, was identified as a covalent binder for the E3 ligase RNF114.^80^ In addition, they also developed a PROTAC that comprised nimbolide and JQ1 (XH2; **Fig. 5D**), and demonstrated that it induced some degradation activity of endogenous BRD4. Zhang et al. also synthesized hetero-bifunctional molecules consisting of a ligand for FKBP12 and three different electrophilic warheads, joined by a PEG linker.^81^ They treated HEK293T cells with those compounds and found that KB02-SLF (**Fig. 5E**) showed moderate degradation activity on FKBP12. Proteomics analysis and a following confirmatory pulldown SILAC (stable isotope labeling by amino acids in cell culture) experiment revealed that KB02-SLF degrades FKBP12 via recruitment of CRL4^DCAF16^. Ohoka et al. also reported a PROTAC that uses a novel E3 ligase.^82^ They chose to focus on the arylhydrocarbon receptor (AhR) E3 ligase and developed PROTACs based on ligands of AhR. β-NF-ATRA (**Fig. 5F**), a PROTAC that directed CRABPs, induced degradation of CRABP1 with AhR dependency, albeit with the fastidious effect of inducing self-degradation of AhR, akin to the mode of action of some SNIPERs.^39–41^

### Target-Based Screening versus Phenotypic Screening Approaches

The exciting progress during the past few years in targeting E3 ubiquitin ligases with more drug-like small molecules, and in using them for developing cell-active PROTAC degraders, motivates the deployment of known, and the development of new, hit- and ligand-finding approaches to screen E3 ligases to discover new chemical matter against them.^83^ HTS against large compound collections is a well-established approach to find hit compounds for “druggable” targets [e.g., kinases, G protein-coupled receptors (GPCRs), and ion channels]. HTS campaigns for finding ligands of E3 ligases are not, however, straightforward. Among the reasons are difficulties to select a proper readout to detect compound activity against a targeted E3 ligase, because the ubiquitination activity of E3 ligases does not provide straightforward readouts that can be monitored in bioassays. Targeting of the substrate binding site of E3 ligases has shown much success for developing inhibitors and PROTACs (e.g., for VHL, CRBN, and IAPs), but that requires the disruption of protein–protein interactions, which are challenging targets for drug discovery.^84^ Moreover, many substrate-binding pockets on E3 ligases and E3 substrate receptor subunits are shallow or highly charged, features that do not make them tractable targets with classical compound libraries. Because of the reasons mentioned above, and because for PROTAC development only binding ligands are needed, regardless of their inhibition activity on their own, other approaches are being developed and applied to find new binders of E3 ligases.

#### Fragment-Based Drug Design (FBDD) Approach

**Figure 6.**
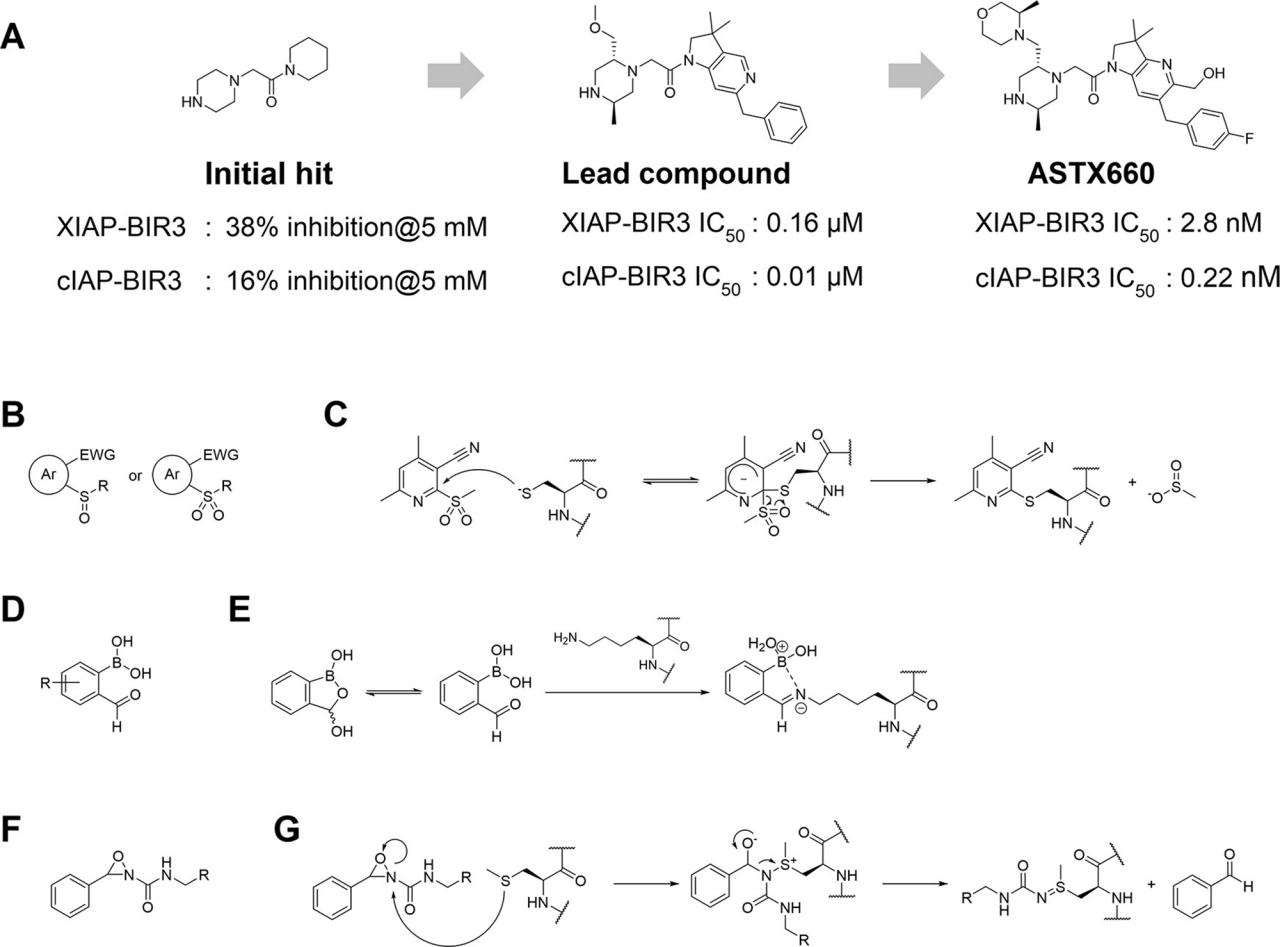
Noncovalent and covalent fragment-based approaches for hit finding and ligand development for E3 ligases. (**A**) Initial hit compound to the final clinical compound, ASTX660.^87–89^ (**B**–**F**) Examples of specific residue-selective covalent warheads: (**B**) cysteine,^150^ (**D**) lysine,^151^ and (**F**) methionine;^152^ and (**C,E,G**) the reaction mechanisms.

E3 ligands for PROTACs are not required to have functional activity in their own right against their targeted ligase. In principle, it suffices that they “bind” to a pocket or surface of the E3 ligase, to provide a suitable starting point for the design of the bifunctional PROTACs. To this end, fragment-based screening is a gold standard method to find new binding pockets and new ligands for proteins. Lucas et al. screened more than 1200 small compounds that complied with the “rule of three”^85^ using differential scanning fluorometry (DSF) against the VHL–ElonginB–ElonginC complex of E3 ligase.^86^ Primary hit compounds were validated by proton nuclear magnetic resonance (^1^H NMR) spectroscopy and X-ray crystal structure analysis, and then they identified three hit compounds that have weak but measurable binding affinity at two pockets distinct from the HIF1α recognition site: one involving the EloC–CUL2 interface, and the other one involving a previously unknown cryptic pocket in VHL. Fragment-based approaches were also applied to find nonpeptidic ligands for substrate recognition sites on E3 ligases. Researchers at Astex Pharmaceuticals conducted fragment library screening over IAPs and identified nonpeptidic hit compounds, even though their binding affinity to IAPs was in the weak millimolar range of dissociation constants.^87^ They next elaborated hit compounds by structure-guided optimization and medicinal chemistry effort, and finally obtained ASTX660, which inhibits cIAP1 and XIAP at nanomolar levels (**Fig. 6A**).^87–89^ ASTX660 is currently in a Phase II trial being investigated for the treatment of advanced solid tumors and lymphomas.

Screening of noncovalent fragment libraries will continue to play a major role in our efforts to find suitable starting points for E3 ligase ligand design. Fragment-based screening with small molecular covalent binders is emerging as an attractive approach too, however, and therefore should provide an alternative and powerful strategy to identify covalent binders at targetable hotspots of E3 ligases. Resnick and coworkers constructed a library of about 1000 small fragments that have acrylamide or chloroacetamide as covalent-binding warheads.^90^ After validating their nonselective reactivity to thiols, they screened them against 10 proteins that have reactive cysteines by liquid chromatography–mass spectrometry (LC-MS) analysis and identified two fragments that bind OTUB2 or NUDT7. In addition, a variety of covalent warheads for reactive amino acid residues were developed (**Fig. 6B–6G**), allowing us to expand the arsenal of covalent-binding small-molecule fragments.^91^ As mentioned before, there are initial examples that PROTACs based on ligands that covalently bind to E3 ligases were shown to have degradation activity for targeted proteins,^65,78–80^ and that means that weak covalent-binding fragments could be applied to develop novel PROTAC degraders. Therefore, these recent updates could help us to identify new covalent E3 ligase binders in the future.

#### Structure-Guided Drug Design Approach

Solving cocrystal structures of E3 ligases with fragment peptides of substrate proteins is also a major strategy to guide the design of small molecular binders of the ligase. A notable success of this approach has been the development of nonpeptidic ligands of VHL, as described in the section titled “VHL Ligands and Applications to PROTACs,” which combined information provided by the binding of the substrate HIF peptide with fragment-based ligand design. Solving more examples of cocrystal structures of other E3 ligases with corresponding fragment substrate peptides will therefore be important.

## SOCS2

Kung et al. reported cocrystal structures of suppressor of cytokine signaling-2 (SOCS2)–ElonginB–ElonginC complexes with 11-residue phosphopeptides of erythropoietin receptor or growth hormone receptor that center their binding on the Tyr426 and Tyr595 regions, respectively.^92^ Superposition of both cocrystal structures revealed essential key interactions of both substrate peptides to SOCS2 and now provide guiding features for designing nonpeptidic small ligands. Phosphotyrosine was found to have a respectable binding affinity for SOCS2 as a zwitterionic amino acid (in the 10^−4^ M range of dissociation constant), suggesting that it could provide a suitable starting point for ligand design.^93^ The poor permeability of the negatively charged phosphotyrosine group will, however, impose some hurdles to be circumvented in their development as cell-active compounds. In addition, a cocrystal structure of suppressor of cytokine signaling-6 (SOCS6) with a substrate peptide was reported.^94^

## KLHL12

Kelch-like protein-12 (KLHL12) forms the CUL3–E3 ubiquitin ligase complex, and it is known to negatively regulate the Wnt-signaling pathway by ubiquitination and subsequent proteasomal degradation against segment polarity protein Dishevelled homolog-3 (Dvl3). Zhao et al. measured binding affinity of several peptide fragments of C-terminal of Dvl3 to KLHL12 by ^1^H−^15^N heteronuclear single quantum coherence (HSQC) NMR analysis and identified the key peptide motif for binding to KLHL12.^95^ They then solved a cocrystal structure of KLHL12 with a truncated peptide of Dvl3 and found important hydrophobic interaction of prolines with KLHL12.

## KLHDC2

Rusnac et al. reported cocrystal structures of Kelch domain–containing protein-2 (KLHDC2), which is one of the BTB E3 ligases and acts as a substrate receptor of the CRL2 E3 ligase complex, with an eight-amino-acid C-end degron peptide of substrate proteins.^96^ Those structures showed that these peptides were bound to the KLHDC2 pocket in a twisted conformation, and the C-terminal diglycine degron motif was fitted in a tight groove with strong coordination of the terminal carboxyl group via three highly conserved residues of KHDLC2.

## GID4

Glucose-induced degradation protein-4 homolog (GID4) is one of the gastric inhibitory polypeptide (GIP) family and part of a GID family that composes the CTLH E3 ligase complex. GID4 recognizes N-terminal proline residues selectively in substrates to mark their ubiquitination and proteasomal degradation,^97^ but the mechanism of molecular recognition remained elusive. Dong et al. reported the cocrystal structures of the globular domain of GID4 with N-end proline fragments and observed that the terminal proline forms key interactions with the residues at the bottom of a deep substrate binding pocket.^98^ In addition, the binding of degrons induces dynamic conformational change around the binding pocket to form hydrophobic interaction with the residues in the pocket.

There are other E3 ligases with reported 3D cocrystal structures containing ligands and proteins that have yet to be exploited for the development of PROTACs (**Table 1**). More structures of E3 ligases with or without bound substrates or small-molecule ligands are expected to be made available in the near future, and they could serve as a starting point for ligand development. Although some ligand-binding pockets seem unsuitable for designing peptidomimetic ligands for PROTACs (e.g., they have a wide and shallow binding area, or a highly charged pocket), a structure-based approach using structural information is one of the most attractive ways to design peptidomimetic ligands for disease-specific E3 ligases and apply them to PROTACs. Toward this goal, our laboratory collaborates as part of a large consortium named EUbOPEN (Enabling & Unlocking Biology in the OPEN), an Innovative Medicines Initiative (IMI)-funded project whose goal is to enable and unlock biology by developing chemical probes and ligands for novel protein classes, including E3 ubiquitin ligases (https://www.eubopen.org). The consortium comprises 22 different partner organizations, including universities, research institutes, European Federation of Pharmaceutical Industries and Associations (EFPIA) members, and small and medium-sized enterprises (SMEs). EUbOPEN will operate under the auspices of a longer-term initiative called Target 2035.^99,100^

**Table 1.** List of E3 Ligases for Which 3D Crystal Structures Are Reported with Bound Ligands or Partner Substrate Proteins, but That Are Yet to Be Explored for PROTAC Applications.

E3 Ligase	Class	Ligand	Pocket	Reference
UBR2	UBR	Peptide	Positive/shallow	^153^
SPOP	BTB	Peptide	Shallow	^154,155^
KLHL3	BTB	Peptide	Negative/shallow	^156^
KLHL12	BTB	Peptide	Shallow	^95^
KLHL20	BTB	Peptide	Negative/shallow	^157^
KLHDC2	BTB	Peptide	Negative/deep	^96^
SPSB1	SPRY	Peptide	Shallow	^158^
SPSB2	SPRY	Peptide	Shallow	^158–160^
SPSB4	SPRY	Peptide	Shallow	^160^
SOCS2	SOCS	Peptide	Negative/shallow	^92^
SOCS6	SOCS	Peptide	Negative	^94^
FBXO4	FBXO	Protein	Shallow	^161^
FBXO31	FBXO	Peptide	Negative	^162^
BTRC	FBXW	Peptide	Negative	^163^
FBW7	FBXW	Peptide	Negative	^164,165^
CDC20	APC/C	Peptide	Shallow	^166^
CDC20	APC/C	Small molecule	Hydrophobic	^167^
ITCH	HECT	Peptide	Shallow	^168,169^
PML	TRIM	Peptide	Shallow	^170^
TRIM21	TRIM	Peptide	Shallow	^171^
TRIM24	TRIM	Peptide	Shallow	^172^
TRIM24	TRIM	Small molecule	Deep	^173,174^
TRIM33	TRIM	Peptide	Shallow	^175^
GID4	CTLH	Peptide	Positive/deep	98

3D: Three-dimensional; PROTAC: proteolysis-targeting chimera.

### Other Target-Based Approaches

HTS is a well-known strategy to identify compounds that bind to target proteins; however, even large compound libraries (e.g., composed of millions of compounds, as available to large pharmaceutical companies) can only cover a tiny area of chemical space. To address this issue, DNA-encoded libraries (DELs) are an attractive combinatorial approach that allows extension of chemical space coverage by small molecules and so is increasingly being applied to drug discovery in academia and pharmaceutical companies.101 One of the most widely applied methods to construct DELs is a split-and-pool approach. This type of DEL usually consists of two to four building blocks, enabling a theoretical single DEL that can have more than a billion compounds in some cases. One of the limitations of DELs is that the libraries can be screened with only cell-free assay systems (e.g., against recombinant purified target proteins); therefore, it is challenging to measure the biological activity of hit compounds directly in a cellular environment. Screening target proteins in vitro means that DELs are a method of choice for the identification of binding ligands, irrespective of their functionality in bioassays or in cells. To this end, DELs are potentially highly suited as an approach to screen for new E3 ligase binders for developing PROTACs. Indeed, several pharmaceutical and protein degradation biotech companies, including Arvinas, Nurix, and Kymera, have disclosed that they are pursuing applications of DEL technologies (either developed as proprietary in house, or via partnerships) to find new E3 ligase ligands. Perhaps curiously, however, no peer-reviewed publication has yet emerged describing the application of DEL technology to identify novel E3 ligase ligands for PROTACs, potentially suggesting specific challenges with targeting the protein surface and protein–protein interaction binding sites that are required to be targeted on E3 ligases. In contrast, novel DELs designed for targeting E3 ligases, for instance covalent DELs,102,103 should provide an attractive approach to discover selective E3 binders and develop novel PROTACs. In fact, some pharmaceutical companies have announced increasing activity and investment to expand the repertoire of E3 ligases for PROTACs by DEL technology.

Phage display technology is one of the greatest ways to obtain peptide ligands for target proteins for which natural substrates and ligands have not been identified.104 In this technology, a randomized synthetic DNA library is inserted into a phage coat protein gene in bacteriophages to express peptides of desired length onto their coat proteins. The obtained “phage library” is incubated with immobilized target (protein, cDNA, antibody, enzyme, small molecule, etc.) and then washed out to remove unbound phages. The remaining phages are eluted and then used to infect bacterial cells to amplify phages for the next cycle. After a few rounds of selection, phages that express high-affinity peptides to the target are accumulated and could be sequenced to identify these peptides. This approach is also suitable to isolate appropriate peptide ligands for developing PROTACs for the same reason as with DELs. It requires additional medicinal chemistry to develop peptidomimetic ligands and improve drug-like properties; however, peptidic PROTACs could be developed rapidly based on peptides identified by phage display as chemical tools to study the recruitment of specific E3 ligases for targeted protein degradation.

In addition to allowing identification of linear peptides, phage display, yeast display, and other display technologies can be used to identify and develop cyclic and macrocyclic peptides too. Cyclic peptides are structurally constrained macrocycles that have emerged as a promising class of molecules to bind to and so target proteins, including targeting protein surfaces and protein–protein interactions in targets once considered unligandable and so undruggable.105,106 Due to their constrained “locked” nature and potential to form intramolecular hydrogen bonds, cyclic peptides can exhibit greater permeability and higher metabolic stability than their linear counterparts, making them attractive as chemical tools to probe novel biology.107,108

Several approaches are being pursued to develop macrocyclic peptides as bioactive or binding/affinity agents. These include rational design,109 DNA-templated synthesis and selection,110 phage-encoded combinatorial display of bicyclic peptides,111,112 and messenger RNA (mRNA) display followed by cyclization.113

Among the mRNA display methods available, the Random Nonstandard Peptides Integrated Discovery (RaPID) system involves peptide spontaneous cyclization and selection, and allows flexible incorporation of nonproteinogenic amino acids. RaPID has demonstrated success at generating cyclic peptide ligands of high binding affinity and specificity for a variety of protein targets.114,115 In an early related application, cyclic peptides were identified targeting E6AP, a HECT E3 ligase target involved in p53-dependent apoptosis.116

### Phenotypic Screening Approach

Molecular glues are attractive starting points for identifying applicable E3 ligases for PROTACs; however, the previously identified molecular glues, immunomodulatory drugs (IMiDs) and sulfonamides, were found serendipitously. For instance, indisulam is a representative compound in the sulfonamide-type molecular glues that was discovered by cell growth inhibitory assay and cell cycle analysis without understanding of its mode of action at that time.117 The major challenge for the discovery of new molecular glues is a limited availability of rational strategies for their identification or design that would induce proteasomal degradation of target proteins. Recently, strategies and approaches have been developed to begin to address these shortcomings. Słabicki et al. analyzed cytotoxicity data of 4518 clinical and preclinical small molecules against 578 cancer cell lines to show correlation between cytotoxicity and expression level of 499 E3 ligase mRNA.118 They found that pan cyclin-dependent kinase (CDK) inhibitor CR8 shows a level of correlation between cytotoxicity of each cell line and the mRNA levels in the same cell line of CDK12 and DNA damage-binding protein-1 (DDB1), which is a component of DDB1–CUL4 E3 ligase complexes. Treatment of CR8 was found to induce degradation of cyclin K without changing the cyclin K mRNA level, suggesting that cyclin K degradation might occur as a result of its ubiquitination via DDB1-associated ubiquitin–proteasome machineries. The cocrystal structure of the cyclin K–CDK12–CR8–DDB1 complex revealed that CDK12 formed extensive protein–protein interactions to DDB1 directly, essentially mimicking interactions formed by DDB1-associated substrate recognition subunits (also known as DCAFs), and that CR8 acted as a molecular glue to enhance this weak-affinity interaction, bringing it to sufficient stability to induce efficient ubiquitination and proteasomal degradation of the CDK12-associated subunit cyclin K. In another study, Mayor-Ruiz et al. performed focused screens in neddylation-impaired cell lines that had the neddylation E2 conjugating enzyme Ube2M knocked out, to bias identification of cytotoxic compounds that were preferentially active and so required neddylation-competent CRL machineries. These screens enabled the authors to find novel chemotypes as hits that were shown to act as molecular glue degraders via DCAF15–CAPERα and DDB1–CDK12–CyclinK, akin to the mode of action of indisulam-like sulfonamides and CR8.119 These reports not only provide a first step toward more systematic approaches to identify small molecules that act as molecular glues, but also expand opportunities to find molecules that hit new E3 ligases in an unbiased manner, in those cases allowing the discovery of molecules that do not interact with the substrate recognition subunit proteins of Cullin RING ligases directly.

Phenotypic screens both provide the opportunity to find molecular glues and can be applied to discover bifunctional PROTAC compounds. For example, anchoring a covalent warhead to a ligand of a target protein provides another way to fish for novel E3 ligases that could be applied to PROTACs, as demonstrated in the case of the studies identifying DCAF16 and RNF4 as described in the section titled “Other E3 Ligases for PROTACs: RNF4, RNF114, DCAF16, and AhR.”79,81 Envisaged extensions of these approaches will involve synthesizing bifunctional molecules that consist of a ligand for a disease-relevant protein, linked to different covalent warheads. Cell lines treated with these compounds and subsequently assessed for degradation of POI represent a strategy to find novel covalently targeted E3 ligases.120

## Future Perspective

Enabled by the discoveries of high-quality, crystallographically defined ligands for the E3 ligases VHL and CRBN, PROTACs have had a meteoric resurgence in the limelight, a remarkable development that has culminated in the first PROTAC degraders being demonstrated as safe and efficacious in the clinic.6,7 These advances have marked a significant step forward and provided added confidence to drug hunters to embark on campaigns to develop PROTAC-based drugs for unmet medical needs, including cancers. It remains challenging, however, to efficiently degrade many proteins using PROTACs. This is because PROTACs need to form stable ternary complexes between the E3 ligase and POI, and PROTAC-induced protein–protein interactions are important to facilitate efficient and selective target ubiquitination and degradation.121,122 It follows that for given target–E3 ligase combinations, it may be challenging to achieve ternary complexes of sufficient stability and suitable properties that make them productive to downstream target ubiquitination and degradation.

The lack of suitable chemical matters for most E3 ligases and undruggable disease targets represents a major stumbling block to our ability to efficiently and effectively navigate degrader drug space. Therefore, there is no doubt that enabling more E3 ligases with new chemistry and new binding ligands becomes an important objective that, when met, will augment the applications and potential of PROTACs to expand in scope to more disease-relevant target proteins. In forecasting novel E3 ligase discovery, we believe that the variety of approaches we have highlighted in this review should help to enrich the armory of E3 ligases that can become amenable to PROTAC drug development. The tremendous progress seen in recent years in technologies such as atomic-level cryogenic electron microscopy (cryo-EM) analysis,123,124 refined protein modeling by next-generation computer-aided design and artificial intelligence,125 and others are expected to accelerate this area of research dramatically. Using disease-, tissue-, and/or organ-specific E3 ligases to develop PROTACs should allow for more cell-type-selective degraders, to aid better understanding of targets’ biology and usher in safer drugs with reduced dose-limiting toxicities. To evaluate protein degradation activities more accurately, however, it is essential to better understand not only the localization of target proteins and E3 ligases but also their dynamics in cells under disease-oriented microenvironments. In addition, there is still a long way to go to develop rational, predictive, a priori strategies to design PROTACs that form stable and cooperative ternary complexes between a target protein and E3 ligase. Nonetheless, comprehensive in silico approaches, for instance molecular dynamics simulation and prediction models by artificial intelligence, aided by enormous computational capability (supercomputers and on-demand cloud computing), are ripe for supporting PROTAC design in the future.

PROTACs represent an emerging modality in drug discovery, as a prominent example of a new class of multispecific drugs that form proximity-induced protein–protein interactions between targeted proteins.126,127 Novel degrader molecules are now being developed hot on the heels of PROTACs, and they differentiate by the mechanism of action or the targets being recruited.128 These approaches include monomeric protein degraders,129,130 autophagy-targeting chimeras (AUTACs),131 biological PROTACs (bioPROTACs),132 lysosome-targeting chimeras (LYTACs),133 autophagosome-tethering compound (ATTEC),134 and others. PROTACs remain frontrunners in the targeted protein degradation space, and their rise to popularity has affected the emergence of other modalities of induced proximity pharmacology, including bifunctional molecules recruiting enzymatic activities beyond E3 ubiquitin ligases, such as protein phosphatases135 and protein kinases.136 It could not be a more exciting time to start finding novel binders for E3 ligases and other unliganded target proteins.
